# Components of service delivery for the detection, care and management of children with cerebral visual impairment: a scoping review

**DOI:** 10.1136/bmjopen-2026-123975

**Published:** 2026-10-15

**Authors:** Lauren R Hepworth, Andrew Riley, Temitope Labinjo, John Ravenscroft, Cathy Williams, Brinton Helliwell, Ffion Curtis, Michelle Maden, Charlotte Croft, Vijay S Gc, Andrew Hill, Ruaraidh A Hill, Shelley Robinson, Catrin Tudur-Smith, Fiona J Rowe

**Affiliations:** 1 Institute of Population Health, University of Liverpool, Liverpool, UK; 2 The Scottish Sensory Centre, Moray House School of Education and Sport, The University of Edinburgh, Edinburgh, UK; 3 Bristol Medical School, University of Bristol, Bristol, UK; 4 VISable, Liverpool, UK; 5 Management School, University of Liverpool, Liverpool, UK; 6 Centre for Health Economics, University of York, York, UK; 7 Mersey West Lancashire Teaching Hospitals NHS Trust, Prescot, UK; 8 Liverpool Centre for Cardiovascular Sciences, University of Liverpool, Liverpool, UK; 9 Specialist Teaching and Learning Sensory Service, Kent County Council, Maidstone, UK

**Keywords:** Health Services, Child, Paediatric ophthalmology, Developmental neurology & neurodisability

## Abstract

**Abstract:**

**Objective:**

To identify and report common elements of cerebral visual impairment (CVI) service delivery across international settings around the detection, management and care of children with CVI.

**Design:**

Scoping review.

**Data sources:**

12 electronic databases from inception, alongside a call for evidence.

**Eligibility criteria for selecting studies:**

English-language studies involving children with CVI, carers or professionals involved in the delivery of services for children with CVI conducted in Organisation for Economic Co-operation (OECD) countries.

**Results:**

108 studies/reports were included, spanning 14 countries. Almost half were published within the last 3 years (2023–2025). Results are presented across six service delivery stages. Several overarching themes were identified, including the requirement of a multidisciplinary team approach with effective collaboration and communication; however, the lack of standardisation of definitions/diagnostic criteria hinders diagnosis and service access.

**Conclusions:**

This scoping review highlights a service landscape for CVI which is characterised by increasing recognition but persistent structural weaknesses which undermine detection, access to and coordination of care and support. Included studies were not appraised for risk of bias, therefore limiting the confidence that can be placed on the conclusions of individual studies, including their implications for policy and practice. No costs/health economic studies were found, highlighting the need for more evidence.Across healthcare, education and habilitation settings, the literature consistently identifies system-level gaps in professional competence, identification infrastructure, information transfer, outcome measurement and service development.

**STRENGTHS AND LIMITATIONS OF THIS STUDY**

- The review presents a comprehensive assessment of the literature on factors influencing cerebral visual impairment service provision and encompasses a range of viewpoints including healthcare, education and family.
- As per scoping review methods, quality appraisal of studies was not conducted; therefore, only scope and direction of study findings are reported.
- The review was limited to studies published in English.

## Background

 Cerebral visual impairment (CVI) is widely recognised as the leading cause of childhood visual impairment in high-income countries, reflecting improved survival following preterm birth and neonatal brain injury alongside advances in neonatal and paediatric care.^1–3^ Unlike ocular visual impairment (OVI), CVI arises from disruption to post-chiasmatic visual pathways and visual processing networks, resulting in highly heterogeneous functional visual difficulties that often coexist with motor, cognitive, communicative and sensory impairments.^4–6^

Despite increasing clinical and research attention, CVI remains conceptually and operationally challenging. A lack of consensus regarding definitions, diagnostic thresholds and classification systems has historically impeded early identification, service eligibility and coordinated care.^2 7^ Although recent international efforts have sought to clarify terminology and establish working definitions, CVI continues to be inconsistently recognised across health, education and rehabilitation systems.^8^ As a result, many children experience delayed diagnosis, fragmented pathways and inequitable access to support.^9 10^

The complexity of CVI necessitates a multidisciplinary and cross-sectoral response. Clinical guidelines and expert consensus consistently emphasise the importance of coordinated input from ophthalmology, paediatrics, neurology, psychology, therapy services, education professionals and families.^11 12^ However, evidence suggests that service delivery frequently occurs in professional silos, with limited mechanisms for systematic collaboration or shared decision-making.^13^ Parents and caregivers are often required to act as primary coordinators of care and interpreters of specialist knowledge, a burden that reflects systemic service failures rather than family preference.^9 14^

While a substantial literature describes clinical features, assessment approaches and individual interventions for CVI, far less attention has been paid to how services for children with CVI are organised, delivered and resourced at a systems level. Existing reviews tend to focus on diagnostic tools or intervention efficacy, frequently highlighting the limited availability of high-quality evidence such as randomised controlled trials (RCTs).^15 16^ In contrast, questions concerning service models, referral pathways, professional roles, equity of access and cost-effectiveness remain under-explored, despite their central importance to policy development and practice implementation.

Understanding how CVI services are structured across healthcare, education and community contexts is, therefore, critical to addressing persistent gaps between identification, diagnosis and meaningful support. Mapping the existing evidence of service delivery can illuminate where systems succeed or fail, identify areas of duplication or omission, and inform the development of integrated, child-centred pathways. This scoping review addresses this need by synthesising the international literature on the detection, care and management of children with CVI, with a specific focus on how services are organised, delivered and evaluated across contexts.

## Methods

This scoping review was protocol-based^17^ and was conducted and written in accordance with the Joanna Briggs Institute methodology for scoping reviews^18^ and the Preferred Reporting Items for Systematic Reviews and Meta-Analyses extension for Scoping Reviews (PRISMA-ScR).^19^

### Identifying relevant studies

Evidence was identified using the following criteria.

#### Population

Children with CVI aged 0–17 years, carers of children with CVI and professionals involved in the delivery of services for CVI. CVI was defined as verifiable visual impairment caused by disturbance of the central visual pathways, whatever the mode of presentation.^2^

#### Concept

Services and interventions to support children with (suspected) CVI and their families, including but not limited to the detection, care and management of CVI, with a focus on how services were organised and delivered.

#### Context

Services delivered in hospital, community, educational and/or home settings. Evidence was limited to Organisation for Economic Co-operation and Development (OECD) countries.^20^

### Information sources

A broad range of sources were considered, including observational and interventional studies, qualitative studies, systematic reviews, service evaluations, audits, doctoral theses, professional guidelines, quality standards and practice recommendations. Study protocols, conference abstracts, editorials and letters were excluded. Studies were restricted to English language publications. An example database search strategy is provided in online supplemental file 1. The following electronic databases were searched: MEDLINE, CINAHL, Embase, APA PsycINFO, Social Care Online, Cost-Effectiveness Analysis (CEA) registry, Health Technology Assessment database, ERIC, British Education Index, Education Research Complete and The Cochrane Library (CENTRAL and CDSR) from their inception to June 2025. The grey literature search included targeted Google Scholar, LENS.org and Web of Science searches, in addition to a UK-focused search including the Health Management Information Consortium (HMIC) and websites of selected relevant organisations, for example, The CVI Society, SeeAbility, CVI Scotland. The research team also put out a call for evidence to clinical and educational communities involved in the care of children with CVI and their families for unpublished relevant material, for example, audits, service evaluations and notice of ongoing research.

### Selection of evidence

Search results were downloaded into EndNote V.21 (Clarivate, Philadelphia, PA, USA)^21^ and de-duplicated before uploading for screening and selection using Rayyan (Rayyan Systems, Cambridge, MA, USA).^22^ Studies were selected by title/abstract screening, followed by full-text screening, by two independent blinded reviewers. Any disagreements on eligibility decisions were resolved through consensus and, where necessary, by discussion with a third reviewer.

### Data extraction and synthesis

A data extraction form was developed and piloted (online supplemental file 2). Extracted data included study and sample characteristics, descriptors of service model (eg, nature of service/test/intervention, mode/frequency/duration of service delivery) and economic data. Extraction was conducted by one reviewer and checked by a second reviewer for accuracy and consistency. Synthesis involved a process of iterative comparison and cross-checking within the data extraction form to build a narrative picture of CVI service delivery and reported gaps.

### Data presentation

Data are presented in text, table and diagrammatic form. Evidence was grouped around different stages of the CVI service delivery pathway, namely: screening and identification; diagnosis and assessment; management and interventions; referrals and professional collaboration; caregivers and families; and researching CVI. This thematic approach emerged inductively from familiarisation with the data and was agreed as an organising framework by discussion with subject experts and PPI representatives. The data were further categorised with reference to study design (eg, systematic reviews, observational studies), populations of interest (eg, patients, professionals, caregivers) and domains of enquiry (eg, healthcare, education, habilitation) and service areas (eg, screening and identification, diagnosis and assessment).

### Patient and public involvement

A project specific stakeholder group was established, comprising individuals with lived experience of CVI or acquired brain injury (ABI), carers of children with CVI and professionals working with individuals with CVI and/or ABI. The role of this group was to support decision-making at all stages of the review. The group was chaired by an individual with lived experience and met six times per year. The professionals included occupational therapists (OTs), orthoptists, vision rehabilitation specialists and qualified teachers of learners with visual impairment (QTVIs).

## Results

In total, 10 278 records were identified: 10 116 from database searches, 816 from citation searches and 162 items from grey literature sources (see figure 1). After duplicates were removed and items screened, 108 reports, studies and grey literature items^1 3–16 23–115^ were included.

**Figure 1:**
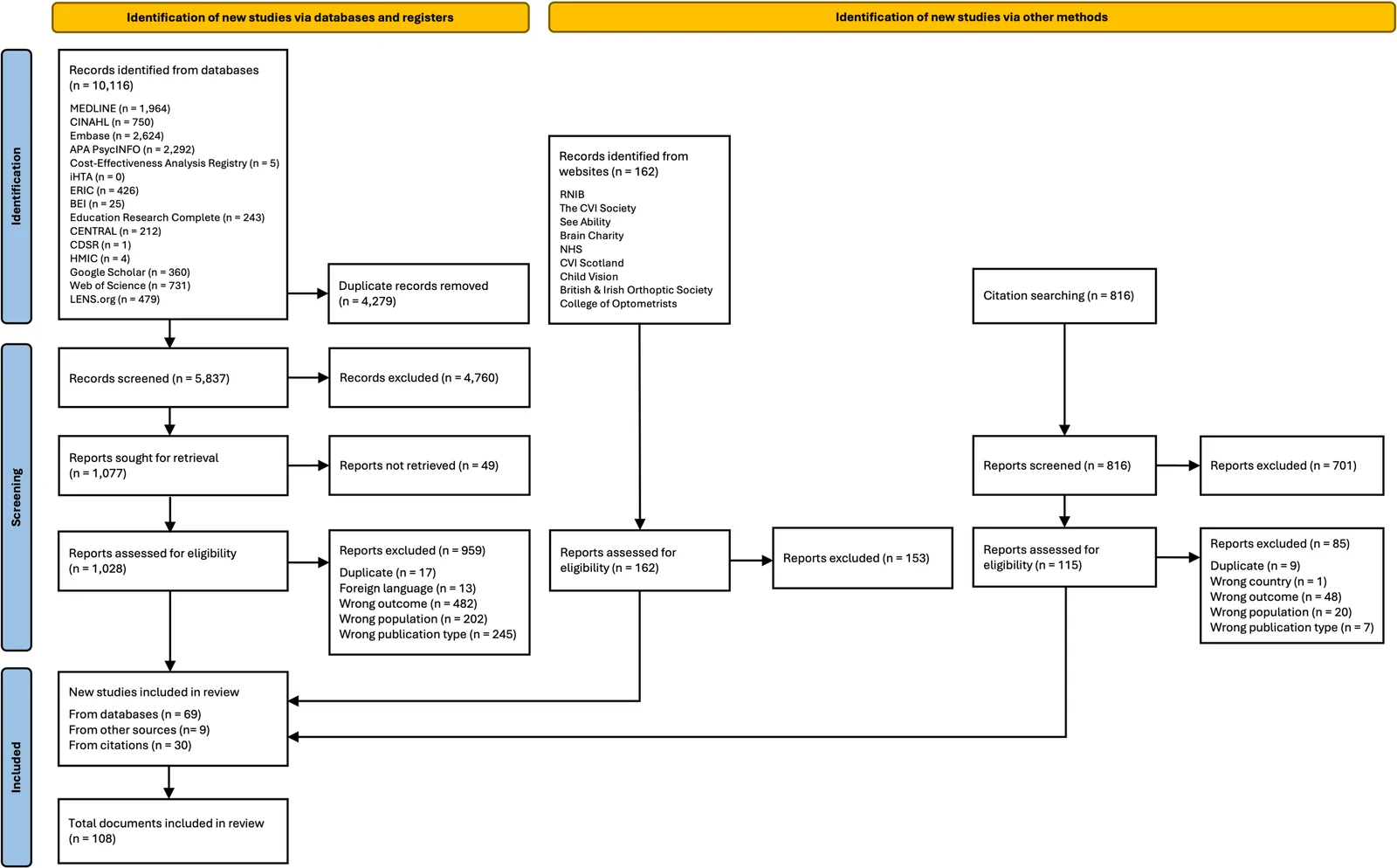
Preferred Reporting Items for Systematic Reviews and Meta-Analyses (PRISMA) flow diagram.^19^

A summary of the included studies is provided in online supplemental file 3. Alongside a brief description of the study aims and methods, the included studies were categorised by study type, overarching domain of enquiry, population of interest and service domain. Online supplemental file 3 also indicates the volume of evidence within each study relevant to each service domain.

### Summary of included studies

Included papers were published between 1987 and 2025. Almost half (n=53) were published between 2023 and 2025, suggesting an accelerating interest in CVI research. Geographically, the majority of studies were conducted in the USA (n=44) and the UK (n=28). Other locations were the Netherlands (n=10), Italy (n=6), Turkey (n=5), New Zealand (n=4), Australia (n=2), Belgium (n=2), Canada (n=1), Israel (n=1), Mexico (n=1), Norway (n=1), South Korea (n=1) and Sweden (n=1), as well as one multi-country study.

While all studies reported issues concerning children with CVI, some focused on subgroups with additional presenting issues, including cerebral palsy (CP),^24 30 32 40 56 82^ OVI,^34^ genetic disorders^51 84 90^ and developmental and multiple disabilities.^83 89^ A small number of studies foregrounded the experiences of professionals supporting children with CVI, including those using augmentative and alternative communication (AAC) modalities,^62 101^ QTVIs/teachers of learners with visual impairment (TVIs)^76 88 106–108^ and learning assistants.^96^

A wide range of document types were identified, including systematic (n=4), scoping (n=3) and literature reviews (n=20); handbooks (n=2); practice guidelines (n=4); policy reviews (n=3); methodological and validation studies (n=5); interventional studies (n=11); observational studies (n=26); case studies/reports (n=13); qualitative studies (n=5); doctoral theses (n=6); and grey literature sources (n=6). The evidence synthesis studies included were helpful in terms of understanding practice gaps and best practice. We found no evaluations of existing real-world services or care pathways for children with CVI, or studies formally taking a systems-wide approach to the topic. Only a small number of randomised studies were identified, addressing heterogenous questions including functional assessment and interventions (eg, three RCTs^46 105 108^), illustrating the dearth of high-quality research into CVI support at a systems level. A greater number of observational studies were included, many of which surveyed professionals and parents/caregivers, or reviewed medical records, to understand experiences of service delivery and use. During data extraction, the collection of economic data (eg, service costs) was planned; however, no relevant data were found.

### Cross-cutting themes

Several overarching themes were identified during cross-comparison of evidence. CVI was widely recognised across the included literature as the leading cause of paediatric visual impairment in high-income countries, often linked to prematurity and conditions like CP. Its impact was often described as profound and systemic, affecting not just vision but also motor, cognitive, language and social-emotional development, learning and participation, and the overall well-being of children.^1 4 6 10 13 14 24 25 29–32 34 50 54 67 85^ Given this complexity, many studies highlighted a lack of standardised definitions, diagnostic criteria, validated assessment tools and management approaches for CVI.^1 7 10 12 13 15 16 31 70 94 95 115^ These inconsistencies were considered to hinder diagnosis, research and service access, underpinning an urgent need for robust clinical and practice guidelines.^7 10 12 13^ Assessing children with complex comorbidities, like multiple-disability, was reported to generate further challenges.^1 4 24 30 32 50^ There was widespread agreement on the necessity of a multidisciplinary team (MDT) approach involving medical (ophthalmology, neurology, paediatrics), allied health (optometry, orthoptics), education (QTVI/TVIs), therapy (OTs, physiotherapists (PTs), speech and language therapists (SLTs), habilitation specialists) and psychology service teams—as well as, crucially, families/caregivers.^1 4–6 10 12–14 24 30 32 50 54 83 85 94 98^ Effective communication and collaboration between these groups were highlighted as vital but often challenging.^5 10 12 30 32 54^ Some studies also highlighted significant evidence gaps; notably, a lack of high-quality evidence (eg, RCTs) supporting the effectiveness of specific CVI interventions, and common methodological weaknesses, such as lack of controls or use of unvalidated measures, among existing literature.^1 10 16 31 70 93 94^

### CVI service domains: gaps and evidence

The review findings were grouped thematically by CVI service delivery stage: (1) screening and identification; (2) diagnosis and assessment; (3) management and intervention; (4) referrals and collaborative practice; (5) families and caregivers; and (6) researching CVI. A logic model (figure 2) illustrating the CVI service delivery pathway was developed inductively from the synthesis of the findings. The model maps the stages of service delivery identified across the literature, highlighting the interactions between healthcare, educational and family support systems.

**Figure 2:**
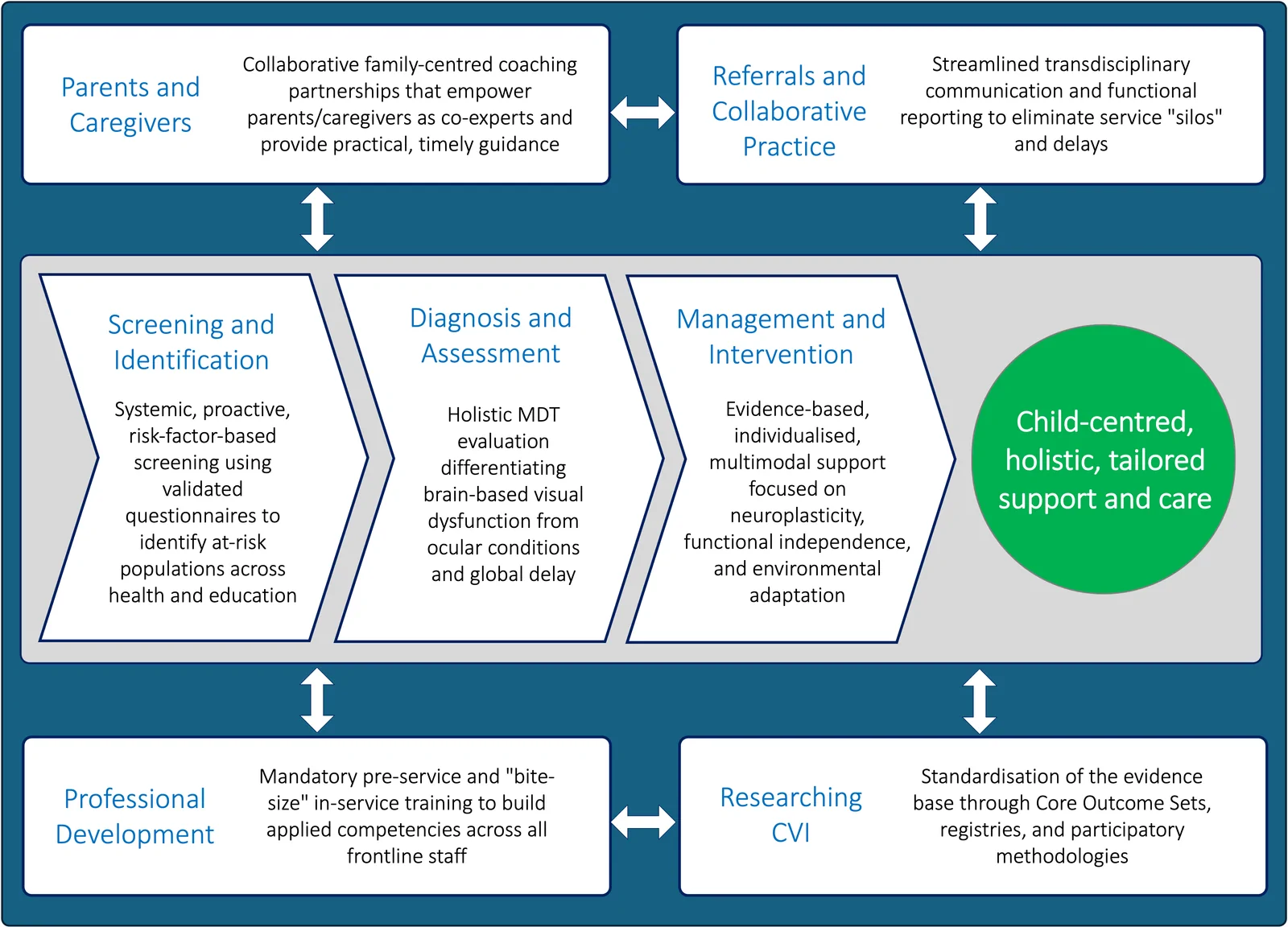
CVI service delivery flow diagram illustrating interlinked service components. The central chevrons show the progression through identification, diagnosis and management towards the goal of child-centred, tailored care. The surrounding panels represent foundational system enablers (parents and caregivers, referrals and collaborative practice, professional development and researching CVI). The bidirectional arrows signify continuous feedback, dynamic interaction and mutual interdependence across all components, highlighting that sustained delivery of optimal care relies on the integration of the entire system. CVI, cerebral visual impairment; MDT, multidisciplinary team.

#### Screening and identification

No description or evaluation of population-level screening programmes was identified. Some studies noted the absence of systematic and routine CVI screening and recommended its adoption.^7 10–13^ A policy paper by Lueck *et al* identified multiple barriers to systematic screening programmes.^13^ These included a lack of consensus around a definition or diagnostic criteria for CVI, narrow ocular/acuity-based classifications of visual impairment that are insufficient for CVI which is a brain-based condition, and the challenge of designing a screening tool that is sensitive and specific enough to differentiate CVI from other neurodevelopmental issues.^13^ It was recommended in several papers that screening or identification be based on children with known risk factors (eg, preterm birth, genetic disorder, malformation or brain injury), focusing on cohorts already engaged with the care system, and a call for the validation of available screening tools, like the Preverbal Visual Assessment (PreViAs) questionnaire, to facilitate screening programmes.^10 13 92^

Recommendations for selective screening were echoed by clinical practice guidelines, which argued for proactive and targeted identification across healthcare and educational sectors.^10 12^ Lehman *et al* highlighted the necessity of equitable access to screening to address disparities in CVI support, particularly among children from minoritised groups, who face a higher prevalence of predisposing conditions such as neonatal complications or developmental delays.^10^ Studies describing screening approaches often focused on the role of individual practitioners rather than mechanisms facilitating systematic population-level screening. In general healthcare settings, primary care clinicians (paediatricians or general practitioners) were advised to manually check CVI risk factors using structured history-taking and systematic behavioural observation.^10 83^ This structured history-taking was facilitated by questioning the parent or caregiver, with clinicians encouraged to seek granular detail regarding previous assessments and specific test types.^83^

Parent/caregiver questionnaires, such as the Flemish CVI Questionnaire, PreViAs and Visual Skills Inventory, were recommended as useful tools, aiding the clinical decision-making process by transforming subjective parental concerns into standardised data points.^11 83^ The efficacy of brief, CVI-specific questionnaires, like Dutton’s five questions, was assessed in two studies which reported specificity >80% in mainstream children; however, there was large variation in sensitivity of 12%–82%.^3 116^ To facilitate screening at scale and overcome the barrier of limited specialist clinic capacity, validated virtual testing protocols were suggested as a feasible and convenient pathway for increasing access to screening through a combination of remote questionnaires and visual acuity tests.^23^

In educational settings, teachers and Special Educational Needs Coordinators served as crucial flagging points.^67 74^ Indicators such as children receiving extra educational help or having a neurodevelopmental disorder were described as useful predictors of underlying visual difficulty.^3^ Consequently, several studies advocated for integrating CVI-specific tools (eg, Austin Assessment, CVI Inventory and/or 11 Questions) into routine national vision screening programmes to proactively detect children in the school environment.^56 65 67 91 117^ However, others argued that an obstacle to such strategies was a severe knowledge and training gap among professionals across both general and specialised care.^9 13^ No existing screening or identification programmes were described or evaluated.

#### Diagnosis and assessment

CVI diagnosis and assessment featured prominently in the included studies, particularly among literature reviews and validation studies. Many framed the issue in terms of a practice gap, in which identification of CVI tended to operate on an ad-hoc basis, often initiated by parental suspicion rather than healthcare professional mandate,^9 52^ contributing to missed and delayed diagnosis.^7 9 43^

Several high-level professional guidelines sought to address this gap. In 2022, Boonstra *et al*^11^ published a comprehensive set of multidisciplinary guidelines for CVI diagnosis and referral, based on a series of systematic reviews, across five distinct topics: (1) medical history and CVI questionnaires, (2) ophthalmological and orthoptic assessments, (3) neuropsychological assessment, (4) neuroradiological evaluation and (5) genetic assessment. The recommendations included a 17-point list of ophthalmological and orthoptic tools to be employed during examination. Similarly, in 2023, the UK’s Royal College of Ophthalmologists (RCO) published a set of *Concise Practice Points*^99^ (based on a review of research and clinical practice^12^), outlining three main diagnostic elements: (1) developmental anomalies or positive risk factors, (2) externally observed or reported symptoms and (3) CVI-related visual dysfunction elicited on examination. Developed as a practical tool for clinicians, the RCO guideline also outlined the responsibilities of ophthalmologists and the broader multidisciplinary eye care team in managing CVI and provided several appendices containing additional resources for professionals and caregivers. In 2024, a working definition of CVI was proposed by Chang *et al*,^8^ developed through expert consultation and sponsored by the US’s National Eye Institute, to address the diagnostic gap. Alongside the definition of being ‘a complex disorder associated with heterogeneous visual dysfunctions arising from brain injury or abnormality’, the authors proposed that the term ‘CVI suspects’ be adopted, based on history and presentation, and that this should allow for early access to support while awaiting a definitive diagnosis.^2 8^

Many studies suggested that diagnosis must involve a verifiable visual dysfunction that is greater than expected by any ocular condition alone.^1 8^ A diagnostic flow diagram developed in the UK by NHS Scotland’s Visual Impairment Network for Children and Young People^100^ qualifies this aim by explicitly indicating that higher-order visual processing cannot be diagnosed without a neurodevelopmental assessment including cognition, motor skills, communication and attention. In addition, the guideline recommends that visual processing difficulties be identified in the context of the whole child, thereby enabling a differentiation between specific visual processing difficulties/CVI and global processing difficulties that include vision.^100^

Many studies highlighted the challenge of navigating the profound heterogeneity of CVI and the coexistence of non-visual motor and cognitive impairments during assessment.^6 30 32^ An MDT and holistic approach were considered best practice by many,^1 5 6 11 30^ typically led by ophthalmologists and paediatricians.^10 50^ During assessment, classifying children by motor function, using, for example, the Gross Motor Function Classification System (GMFCS), was argued as helpful, as non-ambulatory children are at a significantly higher risk for CVI.^56^ For children with high-risk comorbidities like CP, some studies described methodologies to compensate for assessment difficulties.^37 40 56^ In habilitation and educational contexts, functional assessments based on the child’s motor function (again using GMFCS) were recommended^32 56^ to prevent children being labelled as “untestable”.^32^ For non-verbal children, specialised tools like preferential looking tests (PLT) and sweep visual evoked potentials (VEP) were discussed as helping practitioners acquire objective data.^1 4 50 94^ One study suggests a persistent gap between VEP and PLT performance may serve as a valuable biomarker for processing deficits in CVI.^36^ To facilitate accurate assessment, authors suggested that environments during clinical examinations be carefully adapted and controlled, using a quiet, low-stimulation setting and practitioners in simple attire, to isolate visual responses from other stimuli.^32 50^

Multiple studies noted that the diagnostic process is hampered by a lack of validated, standardised assessment tools that can reliably measure the full spectrum of possible CVI-related visual deficits across the severity range.^1 5 13 15 70 94^ Furthermore, while advanced investigations like MRI and VEP provide insights into aetiology, their diagnostic utility for CVI per se is limited as they do not always correlate well with functional visual outcome.^1 13 15^ Functional visual assessment tools administered by QTVI/TVIs were recommended in some studies for incorporation into MDT assessments, such as the CVI Range^36 90^ and the Perkins CVI Protocol,^97 109^ but caution must be taken as the evidence support their measurement properties remains limited. A 2023 study by Pilling *et al*^115^ described the development and validation of the visual behaviours (ViBes) matrix, which aids diagnosis of CVI based on identification of visual dysfunction among children with complex needs. Standardised descriptors were provided to support clinicians and teachers to identify areas of concern, and the matrix demonstrated moderate-to-strong inter-rater reliability.^115^ Although several tools for identifying CVI were examined in the literature, none of the included studies evaluated how they are embedded or utilised within daily clinical or educational practice.

#### Management and intervention

Current CVI management and intervention practices were reported as involving a tension between clinical urgency driven time-sensitive neuroplasticity during early development and the difficulties of accurate diagnosis in very young children, together with the scarcity of robust evidence supporting specific interventions.^4 10 34 54^ Many studies highlighted that there is currently insufficient high-quality evidence to establish an evidence-based treatment pathway specifically for CVI^1 10 13 15 70^ and support many of the interventions that may be employed by clinicians and educators.^15 70 94^ Nevertheless, many studies described adaptive and multidisciplinary strategies aimed at ameliorating the functional impact of visual dysfunction on daily life, learning and development.

Many of the evidence reviews stressed the importance of adaptation and early action. Early intervention was reported as vital because the young brain is most adaptive during critical developmental periods. Consequently, delays in intervention were reported to risk permanent or more severe developmental deficits.^1 4 10 13 29 34 50 54^ Some studies suggested that intensive, structured visual stimulation programmes (often parent-led at home) have been associated with improvements in visual function in some studies, although the evidence base is heterogeneous.^38 63 81^ As such early intervention is frequently advocated on the basis of developmental plasticity. However, the extent to which intervention timing influences long-term outcomes remains uncertain. The literature identifies individualisation as paramount, as interventions must be tailored to the child’s unique profile of visual and non-visual limitations.^1 6 13 30 34 54 68^ Furthermore, it has been suggested that interventions designed for OVI may be ineffective or detrimental for CVI, and people supporting children with CVI should instead focus on compensatory and multisensory strategies, although this has yet to be fully investigated.^1 34^

Regarding the healthcare teams involved in CVI management, their primary responsibility was described in two papers as optimising the biological platform for visual function and neurological development.^1 15^ This foundation is particularly important given studies reporting that while children with CVI often show some degree of age-related or developmental improvement in visual function over time, this is frequently incomplete.^1 10 15 50^ Within this context, the rigorous management of comorbidities was reported by studies as essential for supporting children with CVI. Ophthalmic management to correct refractive errors and accommodation issues was highlighted as frequently under-prescribed for children with CVI, thereby limiting their visual rehabilitation potential.^68 79^ Provision of spectacles, if needed, was emphasised as an essential enabling step that must be initiated before further management specific to CVI can be truly effective.^79 84^ Similarly, the management of neurological comorbidities, such as epilepsy and CP, was reported as critical, given their association with poorer long-term visual outcomes.^53^

The included studies describe a range of intervention modalities for habilitation and functional therapy designed to support children with CVI and enhance their visual capacities. For example, one report indicated that once a child has had their refractive errors corrected, they can be taught ‘active looking’.^29^ This approach involves encouraging a child to increase awareness of their hands and feet by reaching and touching objects, thereby promoting visual-motor integration.^29^ Visual stimulation programmes were also described as beneficial,^63^ as were fixating or searching/scanning exercises performed in simplified visual environments, for instance, using light, high-contrast objects on a plain background.^36 63^

A functional therapy programme, outlined by Salavati *et al*,^82^ was reported as effective for improving daily skills in children with CP, specifically enhancing functional tasks such as eating and self-care while reducing the demand for caregiver assistance. Similarly, a modified vision therapy was validated for strengthening visuo-perceptual skills.^26^ The web-based ‘My CVI’ programme, a psycho-educational tool developed to help children with CVI (typically those with a developmental age between 6 and 14 years) understand their condition, was reported to increase children’s CVI knowledge and reduce feelings of social exclusion.^71^ AAC was also highlighted as a viable approach for managing the communication needs of many children with CVI.^27 62^ AAC systems such as visual displays, tablets or communication boards offer a pathway for children with CVI and associated neurodevelopmental disorders to develop complex language and express choice. However, the literature noted that these systems require careful adaptation to the needs of individual children, and there remains a paucity of research concerning the use of AAC specifically for the CVI population.^27 62^

Environmental management strategies were also prominent in the included studies. These included reducing clutter in home settings, using plain, high-contrast backgrounds in home and educational settings, and using consistent routines to aid object location.^6 24 67 68^ Environmental simplification has been reported to improve the level of independence shown by children, linked to staff training on modification of environments.^57 59 96^ Online resources outlined a range of simple and creative options for creating environments or settings that promote increasing visual awareness by the child, such as colour tents and ultraviolet light.^112 113^

Several studies suggested that visual habilitation should be intensive and initiated early to capitalise on neuroplasticity. Early visual training and environmental adaptation have been linked to improvements in visual acuity.^38 75^ Interventions employing purposeful, structured visual stimulation were also reported to accelerate and enhance visual recovery significantly more than spontaneous, random visual stimulation in the environment alone.^63 81^ Targeted therapies integrating sensory integration training to improve sensory processing, or adapting standard ‘vision therapy’ to accommodate complex needs like developmental delay, have also been reported to improve success.^26 102^ For community-based support, Habilitation VI UK produced a set of quality standards^114^ to ensure that children and young people with visual impairment have access to high-quality mobility and independence training. These standards offer concrete recommendations for practitioners working across sectors.

Educational professionals and specialised learning environments were further identified as playing a vital role in CVI management. In the UK, QTVI/TVIs act as lead professionals with an MDT, responsible for transforming a child’s CVI profile into an adaptive learning plan.^54^ This process facilitates the distinction between providing access to learning and teaching a child the skills of learning to access, to promote autonomy.^54^ A 2024 practice perspective described the development of the Perkins CVI Protocol,^97^ described as a whole-child-focused educational assessment designed to guide practitioners through a structured workflow (including file reviews, parent interviews and direct observations) to identify 16 specific visual behaviours. It is important to note that it is currently not validated.

A central strategy to educator-led CVI management highlighted in the literature was the modification of the environment and curriculum. Within the classroom, it was recommended that educators focus on reducing visual clutter, simplifying screen displays and implementing low-resource adaptations.^65 106^ For reading difficulties such as crowding, interventions involved the use of expanded text or masking to reduce visual complexity, while leveraging the child’s cognitive strengths (such as the ability to use contextual cues) to decode meaningful text.^39^ Furthermore, the evidence suggested that visual presentations can usefully be adapted; for example, by bringing items closer to the child^24 50 68^ and ensuring well-spaced text to manage crowding effects.^1 34 68^ As was the case for screening practices, none of the interventions described were reported as part of real-world services, so we do not yet know how effective they may be in practice.

#### Referrals and collaborative practice

Several studies emphasised that effective assessment and management of CVI was contingent on timely referral and sustained MDT collaboration across diverse professional groups, including neurologists, orthoptists, ophthalmologists, OTs, SLTs and educators.^27 62^ Empirical evidence suggested that an important systemic barrier to effective service delivery was a marked professional knowledge gap across these disciplines. This deficit frequently results in a fragmented system where health and education professionals work in silos,^27 62^ often leaving parents with little meaningful information on diagnosis.^9^ In many instances, the literature describes a ‘parental burden’ whereby caregivers are required to become the primary CVI experts and educators for their child’s entire professional team; a phenomenon documented in both qualitative interviews^44^ and caregiver surveys.^9 52^

Beyond knowledge gaps, a lack of dedicated time within the professional day was cited as another significant barrier to both inter-agency collaboration and the pursuit of ongoing professional development.^35^ Within the educational sector, funding cuts were specifically highlighted as an impediment to providing the necessary specialised support.^74^ A critical point of failure identified in some studies is the transfer of information from healthcare to education. School staff reported significant delays and struggled to translate specialist clinical reports into classroom practice.^74^ This difficulty arises when reports fail to describe the child’s visual profile in functional, accessible terms, such as specific preferences for colour or difficulties with visual clutter, that can be directly applied to learning environments.^74 76^

While the literature highlighted significant systemic barriers, other studies also identify several key facilitators that enhance inter-agency and multidisciplinary collaboration. To improve practice, healthcare professionals and educators prioritised ongoing access to experienced team members and peer support.^62^ Furthermore, maintaining the adoption of new clinical and educational practices was reported to require sustained institutional effort, including consistent resource provision and structured follow-up support.^96^

One paper suggested that interprofessional education (IPE) models act as a major facilitator by training professionals from different disciplines (eg, SLTs, OTs and educators) together.^62^ This shared training develops a mutual understanding of disciplinary roles and fosters the respect necessary for effective collaborative practice.^62^ The literature also indicates that standardised clinical guidelines, such as those published by Royal Dutch Visio, facilitate collaboration by providing a common language and a shared framework for intervention.^41^ Finally, the adoption of functional reporting styles in clinical assessments, which describe a child’s visual profile in accessible, practical terms, was reported as a vital mechanism for ensuring that clinical findings are successfully translated into educational action by school staff.^74 76^

#### Professional development

Several studies highlighted a pervasive knowledge and training gap across the medical, rehabilitation and educational sectors, creating a barrier to effective service delivery.^9 13 43 52^ Empirical data reveal a systemic deficit in the current professional education landscape. For example, a survey of clinical education programme directors, practising optometrists and ophthalmologists in the USA indicated that fewer than half offer formal CVI didactic education, with most instruction limited to only 1 or 2 hours of lecture time.^98^ This limited exposure was associated with low diagnostic confidence among practitioners, contributing to high rates of diagnostic inaccuracies and delayed referrals. One study found that 98% of mainstream primary teachers and 80% of secondary teachers had never heard of CVI,^106^ suggesting an urgent need for targeted professional development and for mandatory, multidisciplinary training proposed in national policy.^13 62^ One study suggests that a failure to address these gaps results in widespread underdiagnosis and missed opportunities for early intervention.^13^

This systemic deficit was also pronounced within the rehabilitation and therapy fields. OTs reported low rates of formal CVI education in their academic programmes, indicating a critical gap in preparing them to manage the functional and visual-motor complexities of the condition.^48^ Even within specialist settings, such as Norwegian paediatric habilitation services, centres frequently judged their own competence regarding CVI as limited.^43^ Furthermore, SLTs, OTs and educators admitted to a lack of skills concerning the integration of CVI with AAC technologies.^62^ Such gaps lead to services being delivered in ‘isolated silos’, which severely compromises coordinated care.^27 62^

To improve service delivery, studies consistently identified a need for streamlined and integrated practice. Recommendations included a call for the urgent inclusion of CVI content in mandatory pre-service training for medical, therapy and education professionals, shifting the focus from general awareness to applied competencies.^13 48^ Some evidence suggested that accessible, ‘bite-sized’ training for classroom teachers and support staff can rapidly increase CVI knowledge and confidence.^96 107^ For instance, specific training programmes have been reported to have helped educators, including QTVI/TVIs choose activities appropriate to a student’s CVI level, thereby increasing measurable ‘looking behaviours’.^107^ Similarly, one study demonstrated that QTVI/TVIs working with clinicians and researchers could integrate medical data (such as neuroimaging) into a child’s individual education plan.^45^ Providing targeted training to learning assistants has also been shown to significantly increase their confidence,^96^ suggesting that training may benefit all frontline support staff. To address the issue of siloed services, IPE models are recommended to foster mutual respect for disciplinary roles, which is critical for effective collaborative practice.^62^

The importance of access to clear clinical guidelines was further outlined in a study by Fonteyn-Vinke *et al*,^41^ which assessed the use of Royal Dutch Visio guidelines among rehabilitation professionals. Participants reported that these guidelines improved their CVI knowledge and self-confidence while enhancing their ability to create tailored interventions. However, the study noted that the overall effect size was modest and that therapists continued to request more concrete intervention suggestions, highlighting the persistent gap in evidence-based management options.^41^ As with screening and assessment, the literature we found on professional development approaches was based on small, often experimental studies rather than evaluations of established services from a systems perspective.

#### Families and caregivers

The CVI service pathway/s or lack of them, have been described to place parents and caregivers in the position of primary knowledge broker and intervention implementer.^9 14^ Several studies suggest that families are frequently the first to suspect a visual processing issue, yet they are routinely provided with very little information by professionals at the point of diagnosis, and several studies described parents as becoming, in effect, ‘educators of the educators’.^9 52^ Regarding condition management, the evidence suggests that efficacy often relies on the parents’ consistent delivery of intensive visual stimulation and environmental adaptations at home, making their involvement a key predictor of positive outcomes.^29 81^

To navigate fragmented service systems effectively, caregivers require timely, practical information and collaborative partnerships with knowledgeable professionals. Studies reported that the most crucial need was access to a trusted professional who can help caregivers interpret, filter and apply complex information directly to their child.^9 34 44 49^ This is a process that empowers families to move beyond a passive medical model and necessitates a collaborative, coaching-style partnership that relieves caregivers of the burden of educating their child’s professional team themselves.^9 34 44 49^ However, such partnerships are contingent on professionals being well-informed and having appropriate resources for partnership-building.^27 52^ Parents reported profound difficulty in obtaining appropriate educational support. One survey indicated that up to 29% of children receive no adjustments due to a lack of CVI-specific training among school staff.^9^ From the family perspective, the literature called for a shift towards a transdisciplinary, coaching-based model that respects the parent or caregiver as a co-expert and provides the knowledge necessary to implement the child’s individualised visual profile.^14 49^

#### Researching CVI to improve future service design and delivery

A small number of the included studies highlighted aspects of research and evaluation that cut across the other service delivery domains. These studies identify a critical need for a more rigorous and internationally cohesive research landscape to bridge the current evidence gap in CVI service delivery, and offer tools to assist in this objective.^13 73 78^ These studies are unified by a call to shift CVI research from isolated, qualitative accounts of promising practices towards standardised, quantitative and participatory methodologies. A key facilitator for this shift is a Core Outcome Set, developed by Pease *et al*, designed to ensure evaluative research remains relevant to family priorities.^73^ This aligns with the implementation of comprehensive registries, recommended by Lueck *et al*,^13^ that record populations served, methods used, and outcomes achieved as a means of monitoring long-term outcomes at scale. Furthermore, the use of digital analytics has been validated as a vital mechanism for gauging public CVI literacy and identifying the specific information-seeking behaviours of the global CVI community, which in turn informs the development of more effective educational resources.^78^ Collectively, these research-focused strategies aim to establish a common language and an empirical evidence base, which are essential for the development of CVI service pathways and the removal of systemic barriers to care.^13 73 78^

## Discussion

This scoping review highlights a CVI service landscape characterised by increasing recognition of the condition but persistent structural weaknesses that undermine effective identification, coordination and support. From over 100 sources, across healthcare, education and habilitation contexts, the evidence points to widespread rather than isolated failures, with implications for professional training, screening or identification infrastructure, information transfer, outcome measurement and service evaluation to allow maturation and improvements.

A prominent report was the extent of the professional competence gap across sectors. Extremely low awareness of CVI among mainstream teachers, alongside self-reported limited competence within specialist habilitation services, represents a significant barrier to timely identification and meaningful intervention.^43 106^ Given the central role of educators, therapists and clinicians in recognising functional visual difficulties and implementing adaptations, this deficit suggests that CVI remains marginal within pre-service education and training pathways. Reliance on optional or post-qualification professional development appears to have been insufficient, supporting calls for the mandatory inclusion of CVI within pre-service curricula for key professional groups, including OTs, QTVI/TVIs and paediatricians.

The review also exposes a clear mismatch between population-level prevalence estimates for CVI-related visual difficulties and the absence of systematic targeted screening infrastructure. Evidence indicating CVI-related visual difficulties in approximately 3.4% of mainstream primary school children contrasts sharply with the lack of national or population-based screening programmes.^3^ However, CVI is an umbrella term: there are wide variations in the nature and severity of visual deficits involved and affected children are not distributed evenly across the population, but concentrated in high-risk groups (^3^ plus many other studies). Correspondingly, where screening or identification occurs, it is largely opportunistic or restricted to high-risk clinical cohorts. However, ad hoc approaches risk inequitable identification and delayed access to support. Without coordinated screening or case-finding strategies embedded across health and education systems, CVI is likely to remain under-identified despite growing awareness.

A further reported systemic weakness lies in the transfer of information between clinical and educational settings. Multiple studies identified clinical reports as a point of failure, frequently lacking the functional descriptors required to support educational adaptation.^74 76^ Reports that focus on diagnosis or test outcomes without describing practical implications, such as sensitivity to visual clutter, colour preferences or environmental triggers, limit teachers’ ability to translate assessment into effective classroom practice. This disconnect undermines the purpose of diagnosis and highlights the need for functionally oriented, education-facing reporting practices.

Traditional reliance on visual acuity as the key measure of visual impairment fails to capture the different visual domains and the functional and participatory impact of visual deficits associated with CVI, as reported in several studies. The development of a Core Outcome Set for paediatric CVI represents an important reframe, prioritising outcomes related to well-being, social interaction and learning.^73^ However, we found limited evidence of systematic adoption of such frameworks in service evaluation, suggesting that their potential to drive meaningful service improvement has yet to be realised.

Parents and caregivers were consistently positioned as primary knowledge brokers within fragmented service pathways. Evidence suggests that inadequate information provision at the point of diagnosis forces families into the role of ‘educators of the educators’, often at significant emotional cost.^9 52^ This reliance on parental mediation may reflect gaps in service coordination and information provision rather than family-centred care in its intended form and underscores the need for clearer communication, coordinated pathways and access to knowledgeable professionals who can support families across sectors. This may also create inequities for children whose families have fewer resources, capacity or opportunities to navigate fragmented services.

Finally, the studies we found indicated that CVI services lack a shared infrastructure. The absence of coordinated data on populations served, distribution of need, interventions delivered and outcomes achieved limits service planning, evaluation and research progression. Emerging approaches, including digital analytics^78^ and condition-specific registries,^13^ offer a pathway toward more coherent, evidence-based service development. Such infrastructure should be viewed as foundational rather than ancillary if CVI is to transition from a niche area of practice to a mature service domain capable of supporting service delivery that offers equitable and sustainable care.

As is appropriate for a scoping review, the included studies were not appraised for risk of bias, and only scope and direction of their findings are reported as published. Therefore, there are limitations to the confidence that can be placed on the conclusions of individual studies, including their implications for policy and practice. No studies with costs or health economics related to CVI services were found and therefore it is not possible to comment on these dimensions except to flag the need for more evidence. The review restricted the inclusion of studies to OECD countries and English-language only, therefore non-OECD and non-English service models (part of Asia, Latin America and Africa) are absent. The grey literature search was largely biased towards UK organisations, despite supplementary searches; for example, Google Scholar. These factors limit generalisability of the review findings beyond high-income Anglophone-centric contexts.

Strengths of this scoping review include that it adhered to a predefined publicly available protocol,^17^ and relevant methodological guidance and reporting guidelines.^18 19^ The included studies are from a range of OECD countries and report a range of viewpoints (medical, educational and family-based). A wide range of studies on CVI services/care are presented and important concepts and gaps in the evidence to date are highlighted by many authors. These include that CVI services are not sufficiently ‘joined up’ and that parents have to face substantial information and coordination burdens for families. There is a lack of information infrastructure such as registries relevant to CVI, there are few well-designed intervention studies and there is a need for studies to harmonise their findings to allow cross-study comparisons and meta-analysis. The scoping review findings set the scene for future primary studies to fill the evidence gaps and for future evidence-synthesis studies to consolidate the evidence base and develop robust policies and practices for the future, to support children with CVI and their families.

## Supplementary material

10.1136/bmjopen-2026-123975 online supplemental file 1

10.1136/bmjopen-2026-123975 online supplemental file 2

10.1136/bmjopen-2026-123975 online supplemental file 3

## Data Availability

Data sharing not applicable as no datasets generated and/or analysed for this study.
