# Supplementary material for: Components of service delivery for the detection, care and management of children with cerebral visual impairment: a scoping review
Source: BMJ Open. 2026 Oct 15;16(10):e123975. doi: 10.1136/bmjopen-2026-123975 (PMC13684197; doi:10.1136/bmjopen-2026-123975)
Supplement: Supplementary file 1 [file bmjopen-2026-123975.reviewer_comments.pdf]

PEER REVIEW HISTORY

BMJ Open publishes all reviews undertaken for accepted manuscripts. Reviewers are asked to complete a checklist review form and are provided with free text boxes to elaborate on their assessment. These free text comments are reproduced below.

ARTICLE DETAILS

Title (Provisional)

Components of service delivery for the detection, care and management of children with cerebral visual impairment: A scoping review

Authors

Hepworth, Lauren; Riley, Andrew; Labinjo, Temitope; Ravenscroft, John; Williams, Cathy; Helliwell, Brinton; Curtis, Ffion; Maden, Michelle; Croft, Charlotte; Gc, Vijay S; Hill, Andrew; Hill, Ruaraidh A.; Robinson, Shelley; Tudur-Smith, Catrin; Rowe, Fiona J

VERSION 1 - REVIEW

**Reviewer**	**1**
**Name**	**Wang, Xing-Hua**
**Affiliation**	**Huazhong University of Science and Technology**
**Date**	**24-Jul-2026**
**COI**	**None**

This is a well-conceived, protocol-driven scoping review mapping how services for children with cerebral visual impairment (CVI) are organised across healthcare, education and habilitation settings in OECD countries. The topic sits squarely within BMJ Open's health-services and public-health remit. The synthesis of 108 sources into six service-stage themes (screening, diagnosis, management, referral/collaboration, families, research infrastructure) is clinically and policy-relevant, particularly the emphasis on systemic rather than isolated failures and the absence of cost/health-economic evidence. The writing is generally clear and the supplementary files (search strategy, data-extraction sheet) support reproducibility. I recommend acceptance after minor revisions to tighten reporting precision and avoid overstatement.

1.The Abstract states "almost half were published within the last three years (2023–2025)" and later "median publication date was 2022" – these are not contradictory but the abstract could state the median explicitly to avoid reader confusion. Also the Abstract conclusion says services are "characterised by increasing recognition but persistent structural weaknesses";

this is fair, but the phrase “no costs/health economics studies were found” should be moved from body to abstract limitations line, since it is a central scoping finding.

2.The manuscript correctly notes no risk-of-bias appraisal was done (appropriate for scoping). However, in the Discussion the authors occasionally drift into phrasing that implies effectiveness judgements (e.g., “early intervention was found to risk permanent deficits”, “intensive visual stimulation accelerates recovery”). These are reported claims from included studies, not conclusions of this review. Please prefix such statements with “Included studies reported that…” or “Primary literature suggests…” to preserve the scoping epistemology and avoid spin.

3.The Methods state the review was limited to OECD and English-language publications. This is acceptable but under-discussed as a structural limit. In the Limitations paragraph, explicitly note that non-OECD and non-English service models (e.g., parts of Asia, Latin America, Africa) are absent, so the “international” claim in the title is effectively “high-income Anglophone-centric”. This does not invalidate the work but should be owned transparently.

4.The logic model is conceptually useful but in the proof the text overlay (“model the interrelation between…”) is broken across pages 66–67 and the diagram itself is not legible as supplied. Please supply a vector/standalone high-resolution figure with a caption that explains each box-arrow relation in plain language; BMJ Open figures must be independently interpretable.

5.“QTVI/TVIs” is introduced but not expanded on first use in the Abstract; spell out “qualified teachers of vision impairment (QTVI) / teachers of vision impairment (TVI)” once. Same for “MDT” (multidisciplinary team) on first appearance in body.

6.Table 1 legend: The symbol key (filled circle = primary focus, empty circle = secondary focus) is clear, but several rows use “√” and “●” interchangeably in the service-domain columns – reconcile to one notation system.

7.p. 13 “CVI is the leading cause…” – add “in high-income countries” (already in Background) to avoid overgeneralisation.

**Reviewer**	**2**
**Name**	**Yap, Tiong Peng**
**Affiliation**	**IGARD Vision Therapy Center**
**Date**	**25-Jul-2026**
**COI**	**None**

This scoping review addresses a crucial and under-researched area - the organisation, delivery, and resourcing of services for children with Cerebral Visual Impairment at a health systems level. The authors highlighted several overarching themes, including the necessity of

multidisciplinary collaboration, barriers to service access, and a lack of standardised diagnostic criteria. The review is highly relevant as it addresses a major gap in the literature regarding how paediatric CVI care is structured, delivered, and resourced internationally. The authors have appropriately adhered to the JBI methodology for scoping reviews and the PRISMA-ScR guidelines, synthesizing a range of document types to identify systemic gaps in care. They have nicely structured the findings across the six service delivery stages which provides a logical and actionable layout for clinicians and policy-makers. While the manuscript purports to cover international CVI service delivery, the grey literature and website searches appear heavily biased toward UK-based organisations. Although the synthesis emphasises a multidisciplinary approach, optometrists seem conspicuously absent from the discussion (citation Ref #98) even though the provision of spectacles is an essential enabling step. The authors also did not mention how accommodative issues can be improved. For example, the authors cited Ref #29 regarding "active looking" but did not mention that special types of spectacles (e.g., near additions, yoked prisms) are usually prescribed alongside visual stimulation activities. It is also interesting that the authors noted specific colour preferences in CVI, but did not elaborate on why or how accommodating to these colour preferences can improve functional vision. Minor comments: Even though the study protocol has been published previously, the authors should still briefly mention how they conducted the search for grey literature. The authors can also elaborate on the nature of Patient and Public Involvement. Please ensure all acronyms are spelled out in full upon their first appearance in the manuscript text (e.g. line 51).

VERSION 1 - AUTHOR RESPONSE

Reviewer 1:
This is a well-conceived, protocol-driven scoping review mapping how services for children with cerebral visual impairment (CVI) are organised across healthcare, education and habilitation settings in OECD countries. The topic sits squarely within BMJ Open’s health-services and public-health remit. The synthesis of 108 sources into six service-stage themes (screening, diagnosis, management, referral/collaboration, families, research infrastructure) is clinically and policy-relevant, particularly the emphasis on systemic rather than isolated failures and the absence of cost/health-economic evidence. The writing is generally clear and the supplementary files (search strategy, data-extraction sheet) support reproducibility. I recommend acceptance after minor revisions to tighten reporting precision and avoid overstatement.

Thank you for the time taken to review our manuscript.

1.The Abstract states “almost half were published within the last three years (2023–2025)” and later “median publication date was 2022” – these are not contradictory but the abstract could state the median explicitly to avoid reader confusion.

The median publication date has been removed from the main text to avoid confusion.

Also the Abstract conclusion says services are “characterised by increasing recognition but persistent structural weaknesses”; this is fair, but the phrase “no costs/health economics studies were found” should be moved from body to abstract limitations line, since it is a central scoping finding.

This sentence has been moved from the conclusions of the abstract, to the strength and limitations bullet points below the abstract.

2.The manuscript correctly notes no risk-of-bias appraisal was done (appropriate for scoping). However, in the Discussion the authors occasionally drift into phrasing that implies effectiveness judgements (e.g., “early intervention was found to risk permanent deficits”, “intensive visual stimulation accelerates recovery”). These are reported claims from included studies, not conclusions of this review. Please prefix such statements with “Included studies reported that…” or “Primary literature suggests…” to preserve the scoping epistemology and avoid spin.

The Results and Discussion sections have been checked for these drifts of phrasing implying effectiveness and have been reworded appropriately. The abstract and strengths and limitations sections have also been checked and rephrased where needed.

3.The Methods state the review was limited to OECD and English-language publications. This is acceptable but under-discussed as a structural limit. In the Limitations paragraph, explicitly note that non-OECD and non-English service models (e.g., parts of Asia, Latin America, Africa) are absent, so the “international” claim in the title is effectively “high-income Anglophone-centric”. This does not invalidate the work but should be owned transparently.

This limitation has been added to the Discussion section.

4.The logic model is conceptually useful but in the proof the text overlay (“model the interrelation between…”) is broken across pages 66–67 and the diagram itself is not legible as supplied. Please supply a vector/standalone high-resolution figure with a caption that explains each box-arrow relation in plain language; BMJ Open figures must be independently interpretable.

We provided an improved image on this submission. The caption has been rewritten to provide further detail about the box and arrow relations.

5.“QTVI/TVIs” is introduced but not expanded on first use in the Abstract; spell out “qualified teachers of vision impairment (QTVI) / teachers of vision impairment (TVI)” once. Same for “MDT” (multidisciplinary team) on first appearance in body.

QTVI and TVI are now defined in full on page 7 within the second paragraph of the summary of included studies section. MDT is defined in full on page 8 within the cross-cutting themes paragraph.

6.Table 1 legend: The symbol key (filled circle = primary focus, empty circle = secondary focus) is clear, but several rows use “√” and “●” interchangeably in the service-domain columns – reconcile to one notation system.

Thank you for indicating that all symbols have not been explained within the legend. We have kept both types of symbols as they are being used for different purposes. However we have now added what the ticks represent within the legend.

7.p. 13 “CVI is the leading cause…” – add “in high-income countries” (already in Background) to avoid overgeneralisation.

Page 13 on the pdf proof reviewed appears to refer to the Cross-cutting themes section. 'developed countries' has been replaced by 'high-income countries' within this section.

Reviewer 2:

This scoping review addresses a crucial and under-researched area - the organisation, delivery, and resourcing of services for children with Cerebral Visual Impairment at a health systems level. The authors highlighted several overarching themes, including the necessity of multidisciplinary collaboration, barriers to service access, and a lack of standardised diagnostic criteria. The review is highly relevant as it addresses a major gap in the literature regarding how paediatric CVI care is structured, delivered, and resourced internationally. The authors have appropriately adhered to the JBI methodology for scoping reviews and the PRISMA-ScR guidelines, synthesizing a range of document types to identify systemic gaps in care. They have nicely structured the findings across the six service delivery stages which provides a logical and actionable layout for clinicians and policy-makers.

Thank you for the time taken to review our manuscript.

While the manuscript purports to cover international CVI service delivery, the grey literature and website searches appear heavily biased toward UK-based organisations.

We acknowledge this is the case despite supplementary searches e.g. Google Scholar. We have added this as a limitation within the Discussion section to improve transparency.

Although the synthesis emphasises a multidisciplinary approach, optometrists seem conspicuously absent from the discussion (citation Ref #98) even though the provision of spectacles is an essential enabling step.

We acknowledge this oversight and have now included optometrists within the list of professional groups involved within the multidisciplinary team within the Cross-cutting themes section. Where reference #98 is specifically mentioned in the Professional development section, it has been clarified that practicing ophthalmologists and optometrists were surveyed.

The authors also did not mention how accommodative issues can be improved. For example, the authors cited Ref #29 regarding "active looking" but did not mention that special types of spectacles (e.g., near additions, yoked prisms) are usually prescribed alongside visual stimulation activities.

It was not possible due to the breadth of this review to give details of all management options; one example was offered which combined spectacles and functional therapy. The aim of the review had a specific focus on service organisation and delivery rather than the specifics of all management options, which is the reason for the omission you have identified.

It is also interesting that the authors noted specific colour preferences in CVI, but did not elaborate on why or how accommodating to these colour preferences can improve functional vision.

Whilst this would be interesting, it was not within the scope of the review to elaborate on the reasons colour preference can improve functional vision in children with CVI. The scoping review by Weden et al (2023) is referenced within our review which highlights the need for higher quality evidence with colour related approaches reported amongst the intervention strategies discussed.

Minor comments: Even though the study protocol has been published previously, the authors should still briefly mention how they conducted the search for grey literature.

The sources used as part of the grey literature search have been added to the Information Sources section of the Methods.

The authors can also elaborate on the nature of Patient and Public Involvement.

A sub-section headed ‘patient and public involvement’ has been added to the methods section, detailing the stakeholder group involved in this review. The sentence earlier in the methods section has been removed.

Please ensure all acronyms are spelled out in full upon their first appearance in the manuscript text (e.g. line 51).

The acronyms on page 8 within the cross-cutting theme section are now defined on this first appearance.

VERSION 2 - REVIEW

**Reviewer**	**2**
**Name**	**Yap, Tiong Peng**
**Affiliation**	**IGARD Vision Therapy Center**
**Date**	**15-Sep-2026**
**COI**	

the authors has addressed the reviewer's comments adequately
