# Supplementary material for: Components of service delivery for the detection, care and management of children with cerebral visual impairment: a scoping review
Source: BMJ Open. 2026 Oct 15;16(10):e123975. doi: 10.1136/bmjopen-2026-123975 (PMC13684197; doi:10.1136/bmjopen-2026-123975)
Supplement: Supplementary file 4 [file bmjopen-16-10-s003.docx]

**Supplementary File 3:** Summary of included studies (n=108). *The ticks indicate which overarching domains are covered within the study; the large, filled circles indicate primary focus for the relevant service domain within each study; the smaller empty circles indicate a secondary focus.*

| **Author, year** | **Location** | **Study focus** | **Study design**  **Sample size and participants** | **Healthcare** | **Education** | **(Re) habilitation** | **Lived experience** | **Screening and**  **identification** | **Diagnosis and assessment** | **Management and intervention** | **Referrals and**  **collaboration** | **Professional development** | **Families and**  **caregivers** | **Researching**  **CVI** |
| --- | --- | --- | --- | --- | --- | --- | --- | --- | --- | --- | --- | --- | --- | --- |
|  |  |  |  | **Overarching Domain** | | | | **Service Domain** | | | | | | |
| Systematic reviews (n=4) | |  |  |  |  |  |  |  |  |  |  |  |  |  |
| Boonstra et al, 2019 ^11^ | Netherlands | Guidelines for assessment, diagnosis and referral for CVI | Systematic review series (n=79 studies) | ✓ |  |  |  |  |  |  |  |  |  |  |
| McConnell et al, 2021 ^7^ | UK | Methods to investigate and diagnose childhood CVI | Systematic review (n=45 studies) | ✓ |  |  |  |  |  |  |  |  |  |  |
| Oh & Kim, 2024 ^70^ | South Korea | Interventions for managing CVI | Meta-analysis and systematic review  (n=5 studies) | ✓ |  |  |  |  |  |  |  |  |  |  |
| Weisser-Pike et al, 2023 ^95^ | USA | Measurement properties and clinical utility of CVI assessments used by  OT practitioners | Systematic review (n=15 studies) | ✓ |  | ✓ |  |  |  |  |  |  |  |  |
| Scoping reviews (n=3) | |  |  |  |  |  |  |  |  |  |  |  |  |  |
| Delay et al, 2023 ^16^ | USA | Scope and nature of interventions implemented for children with CVI | Scoping review (n=23 studies) | ✓ |  | ✓ |  |  |  |  |  |  |  |  |
| Fonteyn-Vinke et al, 2022 ^42^ | Netherlands | Viewing strategies used in school-based tasks among children with and without (C)VI and available interventions | Systematic scoping review (n=30 studies) |  | ✓ |  |  |  |  |  |  |  |  |  |
| Weden et al, 2023 ^94^ | USA | Treatment tools and pathways for paediatric OTs | Scoping review (n=20 studies) | ✓ |  | ✓ |  |  |  |  |  |  |  |  |
| Literature reviews (n=20) | |  |  |  |  |  |  |  |  |  |  |  |  |  |
| Baiardi and Battistin, 2022 ^24^ | USA | Rehabilitative interventions and tools used at the Robert Hollman Foundation | Book chapter / evidence review | ✓ |  | ✓ |  |  |  |  |  |  |  |  |
| Bennett et al, 2025 ^25^ | USA | Describe the effect of CVI on learning access, navigation, and social interaction, and identify compensatory strategies | Opinion and evidence review |  | ✓ |  | ✓ |  |  |  |  |  |  |  |
| Bowman and Vijayalakshmi, 2023 ^29^ | UK | Guidance on identifying, diagnosing and managing CVI | Professional brief | ✓ |  |  |  |  |  |  |  |  |  |  |
| Chang and Borchert, 2020 ^15^ | USA | Current research on methods of visual assessment in children with CVI | Evidence review | ✓ |  |  |  |  |  |  |  |  |  |  |
| Chang and Borchert, 2020 ^31^ | USA | Strategies for evaluation and management of CVI | Evidence review | ✓ |  |  |  |  |  |  |  |  |  |  |
| Ciner et al, 2020 ^32^ | USA | Visual function and visual evaluation strategies in children with CP | Evidence review | ✓ |  |  |  |  |  |  |  |  |  |  |
| Dutton and Bauer, 2019 ^34^ | UK | Best practice for parents and carers to support learning | Book chapter / evidence review | ✓ |  |  | ✓ |  |  |  |  |  |  |  |
| Good et al, 2001 ^1^ | USA | Advances in CVI diagnosis and management in 2001 (updating the earlier study) | Evidence review | ✓ |  | ✓ |  |  |  |  |  |  |  |  |
| Good et al, 1994 ^4^ | USA | CVI diagnosis and management in 1994 | Evidence review | ✓ |  |  |  |  |  |  |  |  |  |  |
| Idil et al, 2021 ^50^ | Turkey | Ophthalmologic approaches to assessing babies with CVI | Evidence review | ✓ |  | ✓ |  |  |  |  |  |  |  |  |
| Keil et al, 2017 ^54^ | UK | Diagnosis, developmental  challenges and outcomes of children with VI | Evidence review | ✓ | ✓ | ✓ |  |  |  |  |  |  |  |  |
| Lehman et al, 2024 ^10^ | USA | Diagnosis and treatment options for clinicians | Clinical guidance / evidence review | ✓ |  |  |  |  |  |  |  |  |  |  |
| McDowell, 2023 ^14^ | New Zealand | Development of a practice framework for supporting children with CVI | Evidence review | ✓ | ✓ | ✓ |  |  |  |  |  |  |  |  |
| McDowell et al, 2024 ^67^ | New Zealand | Nature and impact of visual perceptual difficulties in school-aged children | Evidence review |  | ✓ |  |  |  |  |  |  |  |  |  |
| McKillop & Dutton, 2008 ^68^ | UK | Visual problems experienced by children with brain damage and management approaches | Evidence review | ✓ |  |  |  |  |  |  |  |  |  |  |
| Ortibus et al, 2019 ^5^ | Belgium | Multidisciplinary methods adopted in 3 European centres for identifying and assessing paediatric CVI | Evidence review | ✓ |  |  |  |  |  |  |  |  |  |  |
| Pilling et al, 2023 ^12^ | UK | Detecting and diagnosing CVI in a paediatric ophthalmology setting | Evidence review | ✓ | ✓ |  |  |  |  |  |  |  |  |  |
| Salt and Sargent, 2017 ^83^ | UK | Best practice for assessment of children with developmental disability during outpatient consultation | Evidence review | ✓ |  |  |  |  |  |  |  |  |  |  |
| Waddington and Hodson, 2017 ^93^ | UK | Rehabilitation and habilitation strategies for children with homonymous visual field loss (HVFL) | Evidence review | ✓ |  | ✓ |  |  |  |  |  |  |  |  |
| Zihl & Dutton, 2015 ^6^ | Multi-country | A comprehensive account of the condition including approaches to diagnosis and habilitation | Book / evidence review | ✓ | ✓ | ✓ |  |  |  |  |  |  |  |  |
| Handbooks (n=2) |  |  |  |  |  |  |  |  |  |  |  |  |  |  |
| Buultjens and McLean, 2003 ^30^ | UK | Guidance on collaborative practice for professionals supporting children with CP and VI | Opinion and evidence review | ✓ | ✓ |  |  |  |  |  |  |  |  |  |
| Shaman, 2009 ^85^ | USA | Guidance on team teaching and educational support for children with CVI | CVI manual for professionals |  | ✓ |  |  |  |  |  |  |  |  |  |
| Practice guidelines (n=4) | |  |  |  |  |  |  |  |  |  |  |  |  |  |
| Allen et al, 2023 ^99^ | UK | Clinical assessment, investigation, diagnosis, and initial management of paediatric CVI in eye clinics | Practice points and guidelines | ✓ |  |  |  |  |  |  |  |  |  |  |
| Hayton and Wood, 2022 ^114^ | UK | Habilitation quality standards for mobility independence training and support | Practice guidelines and quality standards |  |  | ✓ | ✓ |  |  |  |  |  |  |  |
| Silverrain, 1987 ^87^ | USA | Educational guidelines and suggestions for teachers | Opinion and practice guidance |  | ✓ |  |  |  |  |  |  |  |  |  |
| VINCYP, 2025 ^100^ | UK | CVI diagnostic pathway incorporating basic and higher visual processing | Practice guidelines | ✓ |  |  |  |  |  |  |  |  |  |  |
| Practice and policy reviews (n=3) | |  |  |  |  |  |  |  |  |  |  |  |  |  |
| Chang and Merabet, 2024 ^8^ | USA | Development of working definition of CVI | Special commentary | ✓ |  |  |  |  |  |  |  |  |  |  |
| Lueck et al, 2023 ^13^ | USA | Barriers to evaluating, diagnosing and caring for children with CVI | Policy review | ✓ | ✓ | ✓ |  |  |  |  |  |  |  |  |
| Pease et al, 2021 ^73^ | UK | Development of a Core Outcome Set for evaluating CVI interventions | Literature review, interviews, Delphi survey | ✓ | ✓ |  |  |  |  |  |  |  |  |  |
| Methodological and validation studies (n=5) | |  |  |  |  |  |  |  |  |  |  |  |  |  |
| Almagati and Kran, 2021 ^23^ | USA | Remote synchronous and asynchronous functional vision assessment | Assessment tool evaluation  n=7 (age-matched sample of children with (n=4) and without CVI (n=3)) | ✓ |  |  |  |  |  |  |  |  |  |  |
| Ferziger et al, 2011 ^40^ | Israel | Interdisciplinary visual assessment questionnaire for children with CP | Assessment tool evaluation  n=77 (41 female children; 36 male) | ✓ |  |  |  |  |  |  |  |  |  |  |
| Kılıç et al, 2024 ^56^ | Turkey | Psychometric properties and application of a questionnaire for school children with CP and CVI | Assessment tool evaluation  n=288 (children with CP) |  | ✓ | ✓ |  |  |  |  |  |  |  |  |
| Pilling et al, 2023 ^115^ | UK | Developing and rating a matrix of visual behaviours in pre-verbal and pre-motor children with CVI | Instrument development and validation | ✓ |  |  |  |  |  |  |  |  |  |  |
| Zatta & Willems, 2024 ^97^ | USA | The Perkins CVI Protocol, an educational assessment tool | Practice perspective report |  | ✓ |  |  |  |  |  |  |  |  |  |
| Interventional studies (n=11) | |  |  |  |  |  |  |  |  |  |  |  |  |  |
| Cemali et al, 2022 ^102^ | Turkey | Effect of a sensory integration intervention on sensory processing and motor skills in infants with CVI and CP | RCT  n=36 (infants with CP and CVI, aged 12–18 months) |  |  | ✓ |  |  |  |  |  |  |  |  |
| Fazzi et al, 2021 ^38^ | Italy | Effect of visual training and environmental adaptations on infants' visual function and development | Pilot clinical trial  n=60 (15 infants with peripheral visual impairment, 15 with CVI, 30 controls) | ✓ |  |  |  |  |  |  |  |  |  |  |
| Fonteyn-Vinke et al, 2024 ^41^ | Netherlands | Rehabilitation guidelines' value and use among vision habilitation and rehabilitation workers | Mixed methods  n=63 (11 therapists; 52 professionals) | ✓ |  | ✓ |  |  |  |  |  |  |  |  |
| Geldof et al, 2015 ^46^ | Netherlands | Assess a functional definition of CVI among preterm infants | RCT  n=172 (105 very preterm/very low birthweight children and 67 controls) | ✓ |  |  |  |  |  |  |  |  |  |  |
| Itzhak et al, 2025 ^105^ | Belgium | Effectiveness of an adaptive individualised game-based rehabilitation on visual function, functional vision, and HRQOL. | RCT  n=77 (33 females, 44 males with suspicion of CVI) |  |  | ✓ |  |  |  |  |  |  |  |  |
| Overbeek et al, 2024 ^71^ | Netherlands | Effects of a computerised psycho-educational programme ‘My CVI’ | Mixed methods (pre-, post-test; follow-up questionnaire)  n=39 (children aged 7 to 16 years) |  | ✓ |  |  |  |  |  |  |  |  |  |
| Salavati et al, 2018 ^82^ | Netherlands | Individual functional therapy programme evaluation for children with CP and CVI | Pre-post-test functional therapy intervention  n=5 (2 boys and 3 girls with spastic CP and CVI) |  |  |  | ✓ |  |  |  |  |  |  |  |
| Tsirka et al, 2020 ^91^ | UK | Evaluating the Insight Inventory (a clinical inventory completed by caregivers) and habilitation strategies | Prospective uncontrolled cohort pilot study n=51 (26 boys, 25 girls with CVI diagnosis) | ✓ |  | ✓ |  |  |  |  |  |  |  |  |
| Wall Emerson et al, 2024 ^107^ | USA | Training regimen for TVIs and its effects on the visual behaviours of students with CVI | Analysis of training programme data  n=18 (TVIs) |  | ✓ |  |  |  |  |  |  |  |  |  |
| Williams et al, 2025 ^108^ | UK | Feasibility of an intervention to improve mental health outcomes for CVI-affected children | Feasibility cluster randomised trial  n=7 (schools), n=979 (children) randomised |  | ✓ |  |  |  |  |  |  |  |  |  |
| Woolvine, 2024 ^96^ | UK | Implementing the ‘colour coded eye’ innovation to support pupils with multiple learning difficulties and CVI | Questionnaires and process evaluation  n=49 (learning assistants/support staff) |  | ✓ |  |  |  |  |  |  |  |  |  |
| Observational studies (n=26) | |  |  |  |  |  |  |  |  |  |  |  |  |  |
| Blackstone et al, 2021 ^27^ | USA | Professionals and parents use of AAC | Survey n=272 (209 school-based professionals and 63 parents) |  | ✓ |  |  |  |  |  |  |  |  |  |
| Bosch et al, 2014 ^28^ | Netherlands | Genetic causes of CVI with low vision | Cross-sectional patient record review  n=309 (children with CVI and visual acuity ≤0.3) | ✓ |  |  |  |  |  |  |  |  |  |  |
| Ely et al, 2024 ^35^ | USA | Teachers' knowledge and skills regarding their roles and responsibilities for supporting learners with CVI | Survey  n=238 (QTVI and mobility specialists) |  | ✓ |  |  |  |  |  |  |  |  |  |
| Friede et al, 2023 ^43^ | Norway | How CVI is identified and assessed at Norwegian paediatric habilitation centres | Online questionnaire  n=19 (rehabilitation centres) |  |  | ✓ |  |  |  |  |  |  |  |  |
| Harpster et al, 2022 ^48^ | USA | CVI education need among OTs and teachers of learners with VI | Survey  n=205 (106 TVIs, 86 OTs, 13 OT assistants) | ✓ | ✓ |  |  |  |  |  |  |  |  |  |
| Imamoglu et al, 2024 ^51^ | Turkey | CVI presentations in infants with neonatal encephalopathy | Case control  n=60 (30 infants/toddlers with neonatal encephalopathy; 30 age-matched healthy children) | ✓ |  |  |  |  |  |  |  |  |  |  |
| Jackel, 2019 ^52^ | USA | Follow-up assessment of parent's experience of CVI diagnosis and support | Survey  n=80 (parents) |  | ✓ |  |  |  |  |  |  |  |  |  |
| Jackel et al, 2010 ^9^ | USA | Parent's experience of CVI diagnosis and support | Survey  n=80 (parents) | ✓ | ✓ |  |  |  |  |  |  |  |  |  |
| Jayasinghe et al, 2025 ^106^ | UK | Current awareness among QTVIs of CVI and strategies to improve CVI awareness and outcomes | Survey  n=111 (QTVIs) |  | ✓ |  |  |  |  |  |  |  |  |  |
| Jimenez-Gomez et al, 2022 ^53^ | USA | Neurologic, developmental and ophthalmologic features of children with CVI | Cross-sectional patient record review  n=249 (individuals spanning 779 patient visits) | ✓ |  |  |  |  |  |  |  |  |  |  |
| Lanners et al, 1999 ^58^ | Italy | Responses to visual stimulation in a cohort of children | Cross-sectional patient record review  n=76 (infants and children up to 4 years of age | ✓ |  | ✓ |  |  |  |  |  |  |  |  |
| Longhin et al, 2020 ^60^ | Italy | Comparing final diagnosis of causes of VI with referral diagnosis | Cross-sectional patient record review  n=92 (children with VI including CVI) | ✓ |  | ✓ |  |  |  |  |  |  |  |  |
| Luo et al, 2022 ^62^ | USA | Professional's experience of delivering services to children who use AAC | Survey  n=262 (209 school-based professionals, 39 community-based professionals, 14 university-based professionals) | ✓ | ✓ |  |  |  |  |  |  |  |  |  |
| Malkowicz et al, 2006 ^63^ | USA | Visual stimulation programmes during rehabilitation | Cross-sectional patient record review  n=21 (children from 13 to 120 months) | ✓ |  | ✓ |  |  |  |  |  |  |  |  |
| Morelli et al, 2020 ^69^ | Italy | Personalised rehabilitation intervention training spatial abilities over 10 years | Case study  n=1 (a child with VI) | ✓ |  | ✓ | ✓ |  |  |  |  |  |  |  |
| Pilling and Ravenscroft, 2024 ^76^ | UK | Referral pathways into sensory services for children with a CVI diagnosis | Online questionnaire  116 responses (UK-based QTIVs) |  | ✓ |  |  |  |  |  |  |  |  |  |
| Rainey et al, 2014 ^77^ | Netherlands | Rehabilitation goals among children with VI | Cross-sectional patient record review  n=289 (16.2% diagnosed with CVI) | ✓ |  | ✓ |  |  |  |  |  |  |  |  |
| Ravenscroft et al, 2021 ^78^ | UK | Access and use of online CVI information | Website analysis via Google Analytics |  |  |  | ✓ |  |  |  |  |  |  |  |
| Rice et al, 2024 ^98^ | USA | Entry-level CVI training among optometrists and ophthalmologists | Online survey  n=382 (48 programme directors and 334 eye care professionals) | ✓ |  |  |  |  |  |  |  |  |  |  |
| Rice et al, 2021 ^79^ | USA | Physician prescribing and rehabilitation referral patterns | Cross-sectional patient record review  n=194 (children diagnosed with CVI) | ✓ |  |  |  |  |  |  |  |  |  |  |
| Roman-Lantzy and Lantzy, 2010 ^80^ | USA | Commonalities in diagnoses and outcomes in children assessed using the CVI Range | Outcome report of a developmental and functional vision evaluation  n=77 (children with ocular or cortical visual impairments) | ✓ |  |  |  |  |  |  |  |  |  |  |
| Sahli et al, 2022 ^81^ | Turkey | Visual habilitation programme evaluation, comparing children with VI due to ocular and cerebral causes | Cross-sectional patient record review  n=130 (children with VI) | ✓ |  | ✓ |  |  |  |  |  |  |  |  |
| Silveira et al, 2023 ^86^ | Australia | Visual impairment and disability prevalence and treatment recorded on the Australian Childhood Vision Impairment Register | Data registry audit  n=132 (children with a primary diagnosis of CVI) | ✓ |  |  |  |  |  |  |  |  |  |  |
| Steendam et al, 2024 ^89^ | Netherlands | Prevalence of CVI and visual functioning in children with profound intellectual and multiple disabilities (PIMD) | Cross-sectional patient record review  n=73 (children with PIMD) | ✓ |  |  |  |  |  |  |  |  |  |  |
| van Genderen et al, 2012 ^92^ | Netherlands | CVI diagnosis facilitators for children with good visual acuity in ophthalmic clinics | Retrospective patient record comparison: n=53 (30 children with good visual acuity and CVI, 23 children referred with a suspicion of CVI | ✓ |  |  |  |  |  |  |  |  |  |  |
| Williams et al, 2021 ^3^ | UK | Prevalence and indicators of CVI-related vision problems in primary schools in England | Cross-sectional survey and clinical assessment: n=2,298 (questionnaire responses); n=248 (children assessed: 152 males, 96 females) |  | ✓ |  |  |  |  |  |  |  |  |  |
| Case studies/reports (n=13) | |  |  |  |  |  |  |  |  |  |  |  |  |  |
| Bilkhu and Fisher, 2021 ^26^ | USA | Modified vision therapy activities in a 5-year-old child with CVI and developmental delay. | Case report  n=1 (4-year-old female) | ✓ |  |  |  |  |  |  |  |  |  |  |
| Chen et al, 2024 ^103^ | USA | Components of professional reflective practice that aid collaboration with children and caregivers | Practice report  n=4 (practitioners, TVIs) |  | ✓ | ✓ |  |  |  |  |  |  |  |  |
| Crossman, 1990 ^33^ | Australia | Case comparison and patterns of recovery among multi-handicapped children | Multiple case study  n=3 (multi-handicapped children) | ✓ |  |  | ✓ |  |  |  |  |  |  |  |
| Emadi & Heidary, 2023 ^36^ | USA | Case history of an infant with a history of prematurity | Case report  n=1 (6-month-old male) | ✓ |  |  |  |  |  |  |  |  |  |  |
| Fellenius et al, 2001 ^39^ | Sweden | Reading strategies including Braille to support children with CVI | Ethnographic observation  n=4 (children with CVI caused by periventricular leukomalacia) |  | ✓ |  |  |  |  |  |  |  |  |  |
| Furze and Phillips, 2018 ^45^ | Mexico | Integration of MRI information into educational planning | Case review  n=1 (5-year-old female) | ✓ | ✓ |  |  |  |  |  |  |  |  |  |
| Groenveld and Leader, 1990 ^47^ | Canada | Experiences of habilitation professionals working with multiply  handicapped CVI children | Case study  n=3 (child neurologist, TVI, developmental psychologist) |  | ✓ | ✓ |  |  |  |  |  |  |  |  |
| Kelley et al, 1993 ^55^ | USA | Orientation and mobility specialists and teacher-consultants role supporting children with VI and severe multiple disabilities | Multiple case study  n=3 (children, one of whom has CVI) |  | ✓ | ✓ | ✓ |  |  |  |  | ⚫ |  |  |
| Lam et al, 2010 ^57^ | UK | 6-year follow-up and interventions for a boy with CVI | Case report  n=1 (9-year-old male) | ✓ |  | ✓ | ✓ |  |  |  |  |  |  |  |
| Lueck et al, 1999 ^61^ | USA | Effects of visual environmental management and visual training on an infant | Case study  n=2 (children with CVI) |  |  | ✓ | ✓ |  |  |  |  |  |  |  |
| Perinelli et al, 2024 ^75^ | Italy | Support pathways to enhance neurodevelopment in a child with developmental and epileptic encephalopathy and CVI | Case study  n=1 (2-month-old female) | ✓ |  |  |  |  |  |  |  |  |  |  |
| Saviola et al, 2018 ^84^ | Italy | Diagnosis and rehabilitation of an infant with CVI | Case report  n=1 (30-month-old female with KCNQ2 encephalopathy and CVI) | ✓ |  | ✓ |  |  |  |  |  |  |  |  |
| Thomas et al, 2024 ^90^ | USA | Parent-implemented assessment and behavioural treatments | Case report  n=1 (child diagnosed with CVI and the SCN2A gene) | ✓ |  |  |  |  |  |  |  |  |  |  |
| Qualitative studies (n=5) | |  |  |  |  |  |  |  |  |  |  |  |  |  |
| Boster et al, 2025 ^101^ | USA | AAC design characteristics favoured by vision professionals for children with CVI and complex communication needs | Focus groups  n=9 (vision professionals e.g., TVIs, speech and language pathologists) |  | ✓ | ✓ |  |  |  |  |  |  |  |  |
| Hartmann and Edstrand, 2024 ^49^ | USA | How caregivers use and find CVI resources in the USA | Semi-structured interviews  n=3 (caregivers) |  |  |  | ✓ |  |  |  |  |  |  |  |
| McDowell and Budd, 2018 ^66^ | New Zealand | Effect of classroom clutter and layout on the learning experiences and behaviour of children | Semi-structured interviews  n=6 (3 TVIs, 3 paraeducators) |  | ✓ |  |  |  |  |  |  |  |  |  |
| Pease et al, 2021 ^74^ | UK | School staff's experience of receiving and using specialist advice to improve support for children with VI | Qualitative interviews  n=9 (2 head teachers, 3 teachers/special needs co-ordinators, 4 teaching assistants) |  | ✓ |  |  |  |  |  |  |  |  |  |
| Smolansky et al, 2024 ^88^ | USA | TVI perspectives on digital learning tools for students with CVI | Semi-structured interviews  n=20 (TVIs) |  | ✓ |  |  |  |  |  |  |  |  |  |
| Theses (n=6) |  |  |  |  |  |  |  |  |  |  |  |  |  |  |
| Faby, 2016 ^37^ | USA | An evidence-based music therapy curriculum for children aged 6 to 16 | Doctoral thesis |  | ✓ |  |  |  |  |  |  |  |  |  |
| Furze, 2022 ^44^ | USA | Understand mothers' perceptions of CVI assessment and education programming | Doctoral thesis / multiple case study (n=3) |  | ✓ |  | ✓ |  |  |  |  |  |  |  |
| Livingston, 2019 ^59^ | USA | Staff ability to support adolescents with CVI and present an individualised support plan | Doctoral thesis / mixed methods |  | ✓ |  | ✓ |  |  |  |  |  |  |  |
| McConnell, 2020 ^64^ | UK | Methods of CVI diagnosis and use of assessment tools in special schools | Doctoral thesis / mixed methods  n=163 (123 parents, 40 teachers) |  | ✓ | ✓ |  |  |  |  |  |  |  |  |
| McDowell, 2020 ^65^ | New Zealand | CVI practice framework for use in education and rehabilitation contexts | Doctoral thesis / mixed methods | ✓ | ✓ | ✓ |  |  |  |  |  |  |  |  |
| Pack-Adair, 2024 ^72^ | USA | Teachers' methods of creating learning environments | Doctoral thesis / mixed methods  n=13 (QTVIs) |  | ✓ | ✓ |  |  |  |  |  |  |  |  |
| Online and journalistic sources (n=6) | |  |  |  |  |  |  |  |  |  |  |  |  |  |
| Charters, 2022 ^109^ | USA | Case management and visual assessment at Perkins CVI Centre | Online article / commentary |  | ✓ |  |  |  |  |  |  |  |  |  |
| Crozier-Fitzgerald, 2025 ^104^ | USA | Informational support and guidance for parents and caregivers | Magazine article |  |  |  | ✓ |  |  |  |  |  |  |  |
| CVI Scotland, 2025 ^110^ | UK | Informational support and guidance for parents and caregivers | Leaflet / handbook |  |  |  | ✓ |  |  |  |  |  |  |  |
| InFocus, no date ^111^ | UK | Informational support and guidance for parents and caregivers | Webpages |  |  | ✓ | ✓ |  |  |  |  |  |  |  |
| Little, 2012 ^112^ | UK | Informational support and guidance for parents and caregivers | Online magazine article |  |  | ✓ | ✓ |  |  |  |  |  |  |  |
| The CVI Society, 2025 ^113^ | UK | Informational support and guidance for parents and caregivers | Webpages |  |  | ✓ | ✓ |  |  |  |  |  |  |  |
