# Supplementary material for: Components of service delivery for the detection, care and management of children with cerebral visual impairment: a scoping review
Source: BMJ Open. 2026 Oct 15;16(10):e123975. doi: 10.1136/bmjopen-2026-123975 (PMC13684197; doi:10.1136/bmjopen-2026-123975)
Supplement: Supplementary file 5 [file bmjopen-16-10-s001.docx]

**Supplementary file 1:** Example search strategy (Medline)
